# Optimizing phage therapy with artificial intelligence: a perspective

**DOI:** 10.3389/fcimb.2025.1611857

**Published:** 2025-05-27

**Authors:** Michael B. Doud, Jacob M. Robertson, Steffanie A. Strathdee

**Affiliations:** ^1^ Division of Infectious Diseases and Global Public Health, Department of Medicine, University of California, San Diego, San Diego, CA, United States; ^2^ Department of Ecology, Behavior & Evolution, School of Biological Sciences, University of California, San Diego, La Jolla, CA, United States

**Keywords:** phage therapy, artificial intelligence, phage specificity, gene discovery, phage engineering, machine learning, synthetic biology

## Abstract

Phage therapy is emerging as a promising strategy against the growing threat of antimicrobial resistance, yet phage and bacteria are incredibly diverse and idiosyncratic in their interactions with one another. Clinical applications of phage therapy often rely on a process of manually screening collections of naturally occurring phages for activity against a specific clinical isolate of bacteria, a labor-intensive task that is not guaranteed to yield a phage with optimal activity against a particular isolate. Herein, we review recent advances in artificial intelligence (AI) approaches that are advancing the study of phage-host interactions in ways that might enable the design of more effective phage therapeutics. In light of concurrent advances in synthetic biology enabling rapid genetic manipulation of phages, we envision how these AI-derived insights could inform the genetic optimization of the next generation of synthetic phages.

## Introduction

The nascent fields of phage therapy, synthetic biology and artificial intelligence (AI) are coalescing at a time in history when antimicrobial resistance (AMR) is increasingly an urgent global health threat (Strathdee et al., 2023). Recent estimates indicate that over the next twenty-five years, 39 million people will die from a multi-drug resistant bacterial infection (Naghavi et al., 2024). Bacteriophage (phage) therapy is emerging as a promising tactic to confront this crisis. Despite the fact that phage were discovered over one hundred years ago and played prominent roles in launching the fields of molecular biology and genetic engineering, clinical applications of phage to treat acute bacterial infections were largely limited to parts of the former Soviet Union and Poland until the past decade, when a series of high-profile case reports ushered in a new era of phage therapy in the West (Strathdee et al., 2023).

In his 2020 commentary published in *Frontiers in Microbiology*, Belgian phage researcher Jean-Paul Pirnay re-imagined phage therapy in the year 2035 (Pirnay, 2020). His vision leverages advances in synthetic biology whereby phage could be generated *de novo* based on genetic sequences of bacterial host isolates without the need to ship bacterial cultures to laboratories for phage matching. In cases where bacterial isolates could not be obtained from patients (e.g., due to antibiotic suppressive therapy), he posited that AI algorithms could be applied to metagenomic data to predict the most likely bacterial sequence to facilitate phage matching. Further, he envisioned a global phage governance platform that would create an efficient, standardized, sustainable and ethical phage supply chain.

How close are we to achieving these realities? In this perspective, we review available literature on emerging advances in AI and synthetic biology that could be used to understand and engineer host specificity and other phage functions, considering the entire phage life cycle (i.e., binding and entry, replication, and lysis). We also consider how AI could be used to mine large datasets for accessory gene discovery and phage genome annotation. Finally, given the need to take phage therapy to scale, we discuss future avenues for research that could further advance the field.

## Understanding phage-host specificity determinants using AI

Identifying infectious phage strains for a given host is essential for phage therapy. However, it is logistically challenging to screen a panel of phage on each clinical isolate, especially when time is of the essence in the treatment of patients with multi-drug resistant bacterial infections. With rapid and inexpensive sequencing increasingly available, matching a potential phage to a target host based on bacterial whole genome or metagenomic data has the potential to accelerate these earliest stages of preparing a therapeutic phage. Recently, several groups have made promising use of AI to achieve strain-level prediction of phage infection from host genome sequences (Boeckaerts et al., 2024; Gaborieau et al., 2024).

Strain-level prediction of infectious phages for a given host genome has been reported for *Klebsiella* spp. (Boeckaerts et al., 2024) and *Escherichia* spp (Gaborieau et al., 2024), whereby shared aspects of these studies reflect the current state of the art. In both cases, machine learning algorithms were constructed using genotypic information as features and large phenotypic datasets (i.e., phage-bacteria infection networks, or PBINs) as outcomes in training data. Both studies made use of pre-existing tools to construct relevant features from genotypic information [e.g., Kaptive (Lam et al., 2022), ECtyper (Bessonov et al., 2021)]. The features predictive of infection were the attachment factors that phages of these genera tend to utilize: surface polysaccharide traits such as capsular K-serotype, lipopolysaccharide (LPS) outer core variations, or O-antigen serotypes. Impressively, both studies encompass genus-level diversity, yet can predict strain-level phage-host specificity.

Interestingly, outer membrane proteins are also frequent attachment factors for phages of *Escherichia* and other spp (Nobrega et al., 2018), yet they were not significantly associated with infection in the dataset analyzed by Gaborieau et al. (2024). Strain-specific amino-acid variation in phage receptor proteins has been shown to modulate phage infectivity (Suga et al., 2021), suggesting that future work may be necessary to develop AI-guided phage matching algorithms for outer membrane protein-targeting phages. One potential way to approach this problem is to use protein structural modeling to predict infection based on interactions between phage receptor variants and phage receptor-binding proteins (RBPs). However, this approach has not yet been evaluated to our knowledge and may be susceptible to false-positive results that accurately predict receptor-RBP binding for phage-host pairs that, for other reasons, do not result in productive phage infection. For instance, in an *in vitro* receptor binding experiment, the phage T4 RBP bound to 85% of the 72 strains in an *E. coli* reference collection, yet T4 phage only formed plaques on 11% of the collection (Farquharson et al., 2021). This indicates that, although receptor-RBD binding may be necessary for infection, it is not sufficient, suggesting that deeper understanding of phage-host interactions downstream from phage attachment may need to be incorporated to improve the accuracy of predictive algorithms.

**Figure 1:**
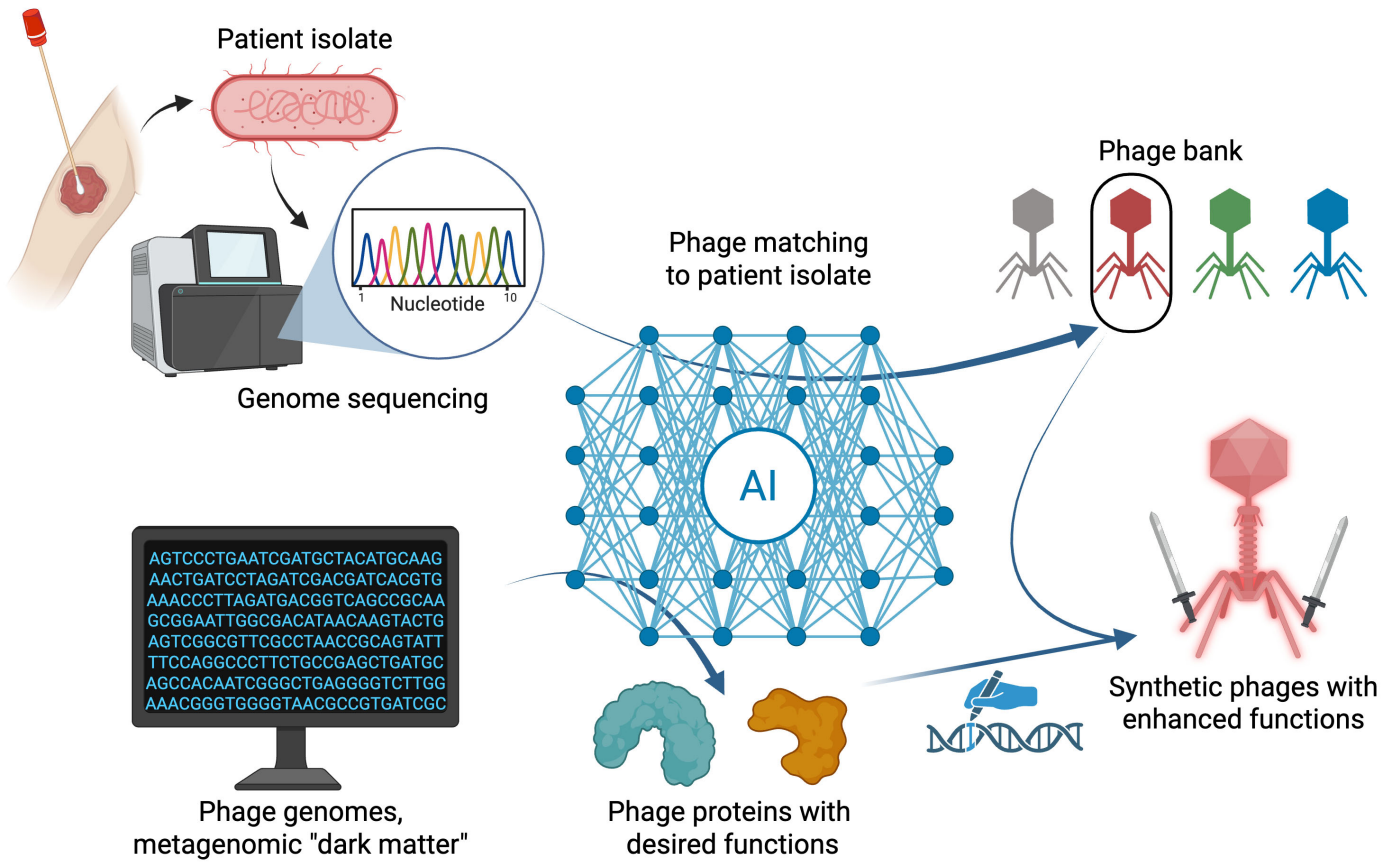
Overview of recent advances and future vision for AI methods to optimize phage therapy. Top: Phage matching based on genetic features in phage and bacterial genome sequences using AI-based algorithms can help identify candidate phages within phage banks for a provided patient isolate bacterium. Bottom: AI algorithms can predict functional phage genes from large sequence databases. Desired phage functions can be genetically grafted onto synthetic phages and evaluated for enhanced phage activity. Created in BioRender.

Even with these recent advances in matching potential phages to target hosts, major gaps remain for strain-level matching in the context of phage therapy. The current AI models for phage matching from host genome sequences are highly specific to host genus, and no classifiers of this type are yet available for highly prevalent ESKAPE pathogens like *Staphylococcus aureus* or *Pseudomonas aeruginosa*. *S. aureus* exhibits surface polysaccharide diversity, with phage predominantly targeting wall teichoic acid (Krusche et al., 2025), so a machine learning approach using teichoic acid variations as features, analogous to using capsular types for *Klebsiella* (Boeckaerts et al., 2024), might be a promising approach. However, for *P. aeruginosa*, phage interactions may involve a larger diversity of receptor types, with resistance mutations in flagella, type IV pili, and LPS evolving against a single phage strain (Kortright et al., 2022). Moreover, some strains of *P. aeruginosa* harbor extensive defense systems (Costa et al., 2024), suggesting these may play a greater role in predicting phage effectiveness from host genomes than that observed in *E. coli* or *Klebsiella*. The degree to which the presence of defense systems is predictive of infection by *Pseudomonas* phages is an area of ongoing work (Müller et al., 2024). With these differences in mind, a holistic approach evaluating the importance of different *P. aeruginosa* genomic features across many phage host pairs (akin to what was undertaken for *E. coli* (Gaborieau et al., 2024) may be required to build a reliable model for this species. Nonetheless, there appears to be great potential for researchers to extend recent examples by leveraging AI to achieve effective strain-level phage matching models in other pathogenic bacteria (**Figure 1**).

Since the above models for phage selection are largely based on host-phage genotype matching, these models would likely struggle to predict the effects of novel resistance mutations outside the training data. Training data are often comprised of a variety of strains of bacteria and phages representing a coarse sampling of genetic variation, but fine genetic variation (such as point mutations) arising during phage-bacteria coevolution can modulate infection outcome. Bacterial evolution of resistance to phage is frequently observed in clinical phage therapy settings (Schooley et al., 2017; Pirnay et al., 2024) and in the laboratory (Luria and Delbrück, 1943; Meyer et al., 2012). Experimental coevolution of bacteria and phages has revealed not only phages’ ability to evolve counter-resistance, but the potential for long-term evolutionary and ecological conflict between phage and bacteria (Borin et al., 2021, 2023; Shaer Tamar and Kishony, 2022; Chen et al., 2024). By experimentally identifying how to position phage with an early evolutionary advantage in the microbial arms race, coevolutionary phage training has the potential to improve the effectiveness of phage therapy (Borin et al., 2021). Recent advances have used machine learning (e.g., L1-penalized logistic regression) to predict the outcome of fine genetic variation in mutation profiles within coevolutionary PBINs assembled through experimental coevolution (Shaer Tamar and Kishony, 2022; Lucia-Sanz et al., 2024). A future challenge will be to combine insights gained through these analyses of fine-scaled genetic variation in laboratory coevolution with predictive models trained on more coarse genetic variation at the strain level observed across natural isolates. Furthermore, while most of the effort in this space has focused on understanding phage-bacteria interactions at the attachment step, which appears to provide the strongest predictive power for infectivity (Boeckaerts et al., 2024; Gaborieau et al., 2024), future work should identify how phage-bacteria interactions downstream of attachment modulate phage activity in ways that could improve efficacy of phage therapy.

## Recent AI advances in the discovery of phage genes with specific functions

There has been an explosion in the number and variety of phage sequences available in public databases in recent years. This has been fueled by both the increase in the number of sequencing studies (often metagenome sequencing) as well as AI-driven improvements in identifying phage sequences in the “dark matter” of metagenome data (Hatfull, 2015). The applications of AI in discriminating phage from non-phage sequences in metagenomic datasets have been extensively reviewed (Nami et al., 2021; Flamholz et al., 2024). There is a wide collection of AI-driven tools for identifying phage genetic sequences (McNair et al., 2012; Auslander et al., 2020; Wu et al., 2021; Johansen et al., 2022; Shang et al., 2023) and classifying phage virion proteins (Thung et al., 2021; Ahmad et al., 2022; Fang et al., 2022). These tools are accelerating the annotation of predicted phage sequences, allowing for a greater diversity of phage sequences to be used for comparative studies.

Ultimately, phage therapy requires cultivated phages, but the discovery of novel functional phage genes in sequence databases can be leveraged to program naturally occurring phages with specific biological functions. A key impediment to harnessing the huge and growing amount of phage sequencing data is that the number of potential genes of interest is vast, making it experimentally intractable to functionally screen sequences for biological function. Several recent studies (Concha-Eloko et al., 2024; Zhang et al., 2024; Yirmiya et al., 2025) exemplify recent advances in leveraging AI to sift through large sequencing datasets to predict putative phage genes with specific functions, triaging labor-intensive experimental validation for predicted candidate genes. Once validated, these novel gene sequences can be tested for their ability enhance the efficacy of engineered therapeutic phages (**Figure 1**).

Anti-phage defense systems are diverse and heterogeneously distributed throughout bacteria (Doron et al., 2018; Bernheim and Sorek, 2020; Georjon and Bernheim, 2023), and there is a growing collection of phage genes that have been identified as counter-defenses against these bacterial immune systems (Vassallo et al., 2022; Mayo-Muñoz et al., 2024; Murtazalieva et al., 2024; Yirmiya et al., 2024). It is likely that many phage counter-defenses are yet to be discovered. Yirmiya et al. (Yirmiya et al., 2025) used protein structure and interaction prediction using AlphaFold2-Multimer (Evans et al., 2022) to screen approximately two million phage genomes containing over 30 million phage genes, and identify phage proteins predicted to fold and interact with pre-chosen bacterial phage defense proteins. By carefully and iteratively designing a computational workflow, they were able to attain a ~50% success rate in identifying experimentally validated novel phage inhibitors of several well-characterized bacterial defense systems. Although there are limitations to this approach – including the need to select appropriate protein-binding partners such as a bacterial defense system of interest *a priori*, and a substantial false-positive rate – this approach more generally establishes a paradigm that leverages AI to predict novel phage genes that interact with specific proteins of interest. As additional bacterial anti-phage defense systems are functionally and structurally characterized, this approach will likely uncover additional novel phage counter-defense genes that may find utility in synthetic phages armed to match the capabilities of the target bacteria they are deployed against.

Bacterial capsules and biofilms are virulence factors that pose challenges in the treatment of bacterial infections (Chang et al., 2022). Some phages rely upon recognition and digestion of polysaccharide components in bacterial capsules and biofilms as their first step in the infection process (Knecht et al., 2020). The use of phage as a strategy for overcoming biofilms in difficult-to-treat infections has long been proposed and is recognized to require very specific interactions between phage and host (Hughes et al., 1998; Mayorga-Ramos et al., 2024). Some phage genes necessary for biofilm and capsule degradation have been identified as depolymerases that can attach to the distal tips of phage tails where they simultaneously act as enzymes that degrade polysaccharide components and as specific receptor-binding proteins for which presence of the cognate capsule is required for infection (Dunstan et al., 2021). Mirroring the genetic and antigenic diversity of capsular polysaccharide serotypes in pathogenic bacteria (Shu et al., 2009), phage depolymerase sequences are also quite diverse in amino-acid sequences (Knecht et al., 2020) and this high degree of sequence variability complicates the process of identifying novel depolymerases in sequence databases. To overcome these difficulties, Concha-Eloko et al. (2024) demonstrate how a protein language model fine-tuned for depolymerases and trained on carefully curated data has advanced the annotation of depolymerase genes and their respective enzymatic domains, beyond currently available computational tools. The improved AI-guided annotation of depolymerase genes enables further study of the use of diverse depolymerase genes in recombinant phages to reprogram specificity and enzymatic capabilities for targeted therapeutic applications against specific capsule types.

Phage lysins are enzymes that degrade peptidoglycans, playing an essential role in the phage life cycle by promoting host cell lysis and cell death. There has long been interest in developing recombinant lysins as treatments for bacterial infections since they can also lyse the cell from the outside (Fischetti, 2018). A recent Phase 3 clinical trial of a lysin targeting *Staphylococcus aureus* added to standard of care antibiotics was ended for futility after an interim efficacy analysis (ClinicalTrials.gov ID NCT04160468). However, there is potential to advance their use, using engineered lysins selected from combinatorial libraries recombining portions of known lysin sequences (Gerstmans et al., 2020), with the potential to develop novel synthetic lysins with fine-tuned specificity and activity. Further advances in lysin engineering – whether for use as therapeutic protein products or as genetic cargo in engineered therapeutic phages – has been limited by the lack of computational methods to comprehensively screen metagenomic or uncharacterized phage genome sequence data to identify new lysin genes. Recent work by Zhang et al. provides a machine learning based software package that identifies putative lysin genes from assembled contigs (Zhang et al., 2024). Among 17 predicted novel lysin sequences selected for experimental validation, seven exhibited appropriate activity. Similar to the approach used by Yirmiya et al. (2025), there is a substantial false positive rate requiring rigorous experimental validation of AI-produced screening candidates, however, these are substantial advances in that they allow researchers to triage valuable time and resources validating candidate genes selected from otherwise intractably large datasets.

A key theme emerging from each of these studies is that an enormous amount of careful human planning, intuition of biological plausibility, and iterative human-driven improvement to AI algorithms is necessary to realize the potential of these approaches. By facilitating a computational screening process for specific biological functions, these emerging AI models are enabling researchers to exploit vast troves of data to discover a diversity of new phage genes that can antagonize bacterial defense systems, degrade biofilm and lyse infected cells, each of which may be useful in the future design of therapeutic phages with desired functions.

## Future outlook: AI-guided development of synthetic phages as enhanced therapeutics

The AI-driven advances described above are beginning to generate tools that can predict which naturally occurring phages are most likely to infect a target bacterium. A deeper level of understanding of which genetic determinants drive these predictions, and the underlying mechanisms behind productive infection, are beginning to emerge to enable phage specificity programming (Dunne et al., 2021). Synthetic biology methods are already available to modify many phage genomes (Jaschke et al., 2012; Ando et al., 2015; Kilcher et al., 2018; Pires et al., 2021; Adler et al., 2022; Assad-Garcia et al., 2022; Kamata et al., 2024) and have begun to be applied to study granular determinants of phage specificity through high-throughput mutational studies of phage receptor binding proteins (Dunne et al., 2019; Yehl et al., 2019; Andrews and Fields, 2021; Huss et al., 2021, 2023). Huss et al. have recently developed a method of analyzing deep mutational scanning data of a phage receptor binding protein to develop a motif-searching algorithm that identifies novel phage receptor-binding sequences from metagenomic data (Huss et al., 2024). Collections of new receptor-binding protein sequences from these and other studies can be used as the substrate for future AI-guided protein design, leveraging generative models of protein sequences (Hsu et al., 2024). In a manner analogous to using protein language models to accelerate directed evolution of antibody sequences targeting specific antigens (Hie et al., 2023), phage receptor-binding protein engineering may also be amenable to machine-learning-guided directed evolution to modulate receptor specificity. While such fine-tuning of phage receptor binding through protein design has the potential to generate phages with defined bacterial receptor targets, it is important to note that binding affinity alone is not always sufficient to confer productive infection (Farquharson et al., 2021), and more work is needed to understand the mechanisms of infection immediately downstream of receptor binding, which are incompletely understood for even some of the most heavily studied model phages (Hu et al., 2015; Ge and Wang, 2024). More broadly, generative models of entire genomes, including phage genomes, have recently been described (Nguyen et al., 2024; Shao and Yan, 2024), and although these models are in their infancy and do not yet produce biologically functional whole genomes, they are already able to recapitulate coarse genomic architectures similar to natural phage genomes and can even produce gene sequences for functionally active multicomponent systems (Nguyen et al., 2024).

The recent AI-guided advances outlined above identifying novel phage lysins, depolymerases, and counter-defenses to bacterial immune systems similarly lay the groundwork for incorporating these functions into designed, synthetic phages (Lenneman et al., 2021) or other therapeutics, expanding the armamentarium of engineered phages that can deliver heterologous effector proteins (Du et al., 2023; Gencay et al., 2023) or augment natural phage function in other ways favorable for therapy. Phages engineered to avoid lysogeny have already been used clinically (Dedrick et al., 2019). Future work will be necessary to identify whether the rational design of synthetic phages with other various functions can increase treatment efficacy. Additionally, the bioethical and environmental implications of treating patients with genetically engineered phages requires continued careful contemplation from a One Health perspective, since engineered phages have the potential to impact human-, animal-, and environment-associated microbial communities (Hernando-Amado et al., 2019; Nair and Khairnar, 2019; Banerjee and van der Heijden, 2023). Although the dream of instant AI-designed therapeutic phage synthesis for a provided target bacterium is unlikely to be achieved in the next 10 years (Pirnay, 2020), advances in AI are both accelerating the identification of naturally occurring phages for therapy, as well as enabling the distillation of useful knowledge from otherwise untenably large sequencing databases abundant with uncharacterized phage genes that could find utility in synthetic phages. In the meantime, coordinated efforts are needed to make the growing number of phage libraries across the world compatible with one another, and accessible for compassionate use cases, clinical trials, and translational research experiments.

## Data Availability

The original contributions presented in the study are included in the article/supplementary material. Further inquiries can be directed to the corresponding author.
